# Monocyte proinflammatory phenotypic control by ephrin type A receptor 4 mediates neural tissue damage

**DOI:** 10.1172/jci.insight.156319

**Published:** 2022-08-08

**Authors:** Elizabeth A. Kowalski, Eman Soliman, Colin Kelly, Erwin Kristobal Gudenschwager Basso, John Leonard, Kevin J. Pridham, Jing Ju, Alison Cash, Amanda Hazy, Caroline de Jager, Alexandra M. Kaloss, Hanzhang Ding, Raymundo D. Hernandez, Gabe Coleman, Xia Wang, Michelle L. Olsen, Alicia M. Pickrell, Michelle H. Theus

**Affiliations:** 1 Department of Biomedical Sciences and Pathobiology, Virginia Tech, Blacksburg, Virginia, USA.; 2 Department of Pharmacology and Toxicology, Faculty of Pharmacy, Zagazig University, Zagazig, Egypt.; 3 School of Neuroscience, and; 4 Translational Biology Medicine and Health Graduate Program, Virginia Tech, Blacksburg, Virginia, USA.; 5 Center for Engineered Health, Virginia Tech, Blacksburg, Virginia, USA.

**Keywords:** Neuroscience, Neurological disorders

## Abstract

Circulating monocytes have emerged as key regulators of the neuroinflammatory milieu in a number of neuropathological disorders. Ephrin type A receptor 4 (Epha4) receptor tyrosine kinase, a prominent axon guidance molecule, has recently been implicated in the regulation of neuroinflammation. Using a mouse model of brain injury and a GFP BM chimeric approach, we found neuroprotection and a lack of significant motor deficits marked by reduced monocyte/macrophage cortical infiltration and an increased number of arginase-1^+^ cells in the absence of BM-derived *Epha4*. This was accompanied by a shift in monocyte gene profile from pro- to antiinflammatory that included increased *Tek* (Tie2 receptor) expression. Inhibition of Tie2 attenuated enhanced expression of M2-like genes in cultured Epha4-null monocytes/macrophages. In *Epha4*-BM–deficient mice, cortical-isolated GFP^+^ monocytes/macrophages displayed a phenotypic shift from a classical to an intermediate subtype, which displayed reduced Ly6c^hi^ concomitant with increased Ly6c^lo^- and Tie2-expressing populations. Furthermore, clodronate liposome–mediated monocyte depletion mimicked these effects in WT mice but resulted in attenuation of phenotype in *Epha4*-BM–deficient mice. This demonstrates that monocyte polarization not overall recruitment dictates neural tissue damage. Thus, coordination of monocyte proinflammatory phenotypic state by Epha4 is a key regulatory step mediating brain injury.

## Graphical abstract

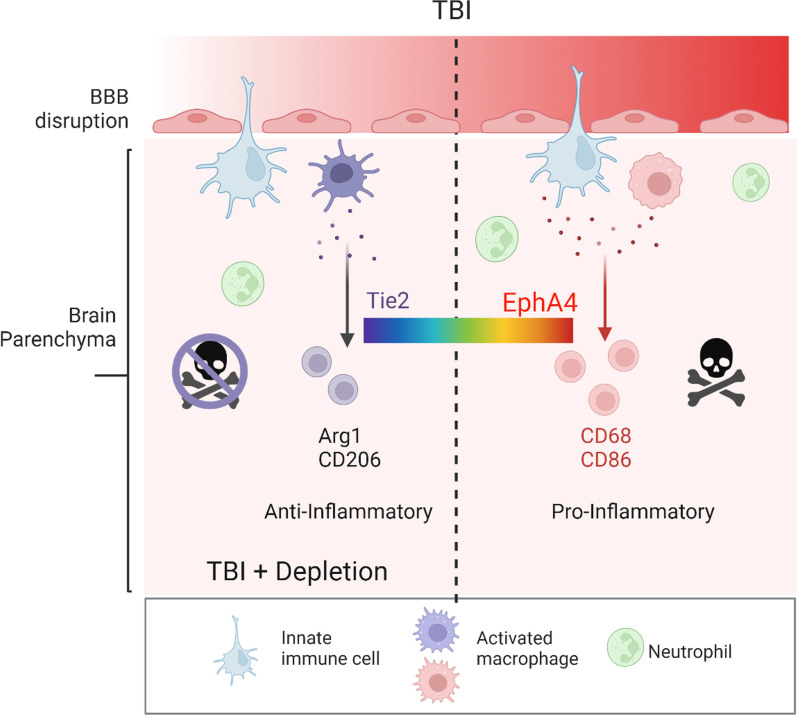

## Introduction

Neuroinflammation is a major driver of secondary damage following brain injury (1). Peripheral immune-derived monocytes have emerged as key players in the exacerbation of neuroinflammation (2) and the pathogenesis of neurological insult (3–6). As essential components of innate immune response, peripherally derived monocytes infiltrate injured tissues and subsequently become activated to macrophages, which can then polarize to functionally distinct subtypes. Historically, monocyte-derived macrophages have been classified simply as having M1 (classical) or M2 (alternative) phenotypes. Classically activated macrophages produce proinflammatory cytokine and matrix metalloproteinases to break down the cytoskeleton, while alternatively activated macrophages produce antiinflammatory cytokines and other remodeling factors to resolve inflammation (7–9). Nonetheless, recent studies suggest that macrophage activation produces a variety of phenotypes in vivo due to their phenotypic plasticity and the complexity of their function (10).

Erythropoietin-producing hepatocellular carcinoma (Eph) receptors compose the largest family of receptor tyrosine kinases; Eph receptors are subdivided in 2 classes, EphA and EphB (11). EphA receptors contain a single transmembrane fragment and several cytoplasmic domains, while EphB receptors are GPI-anchored proteins. The extracellular domain of the Eph receptor interacts with its membrane-bound ephrin ligands, and their binding induces bidirectional signaling which has been implicated in multiple physiological and developmental processes (12). Ephrin type A receptor 4–dependent (Epha4-dependent) axon guidance in development has been extensively investigated; however, its importance is under intense investigation in neurological disorders, including traumatic brain injury (TBI) (13, 14), Alzheimer’s disease (15), amyotrophic lateral sclerosis (16), and ischemia (17).

We recently uncovered a role for Epha4 in the regulation of the function of Tie2, a tyrosine kinase receptor, in endothelial cells and neuroinflammation after brain injury (14, 18), the molecular mechanisms of which may include p-Akt crosstalk. This study uncovered a potentially novel role for Epha4/Tie2 interaction in regulating monocyte recruitment and polarization in neural tissue damage. Furthermore, we implicated Tie2 in suppressing the proinflammatory state of monocytes that is negatively regulated by Epha4 and identify this as a primary mode of controlling the phenotypic state of monocytes.

In this investigation, we utilized a BM chimeric approach to generate *Epha4*-BM–deficient mice and clodronate monocyte depletion to study the relevance of Epha4 for monocyte recruitment and subset polarization in a murine model of controlled cortical impact (CCI) injury. The role of Epha4 on Tie2-expressing monocytes was further interrogated. We conclude from our overall findings that, the polarization state, not the overall influx of monocytes, is central to tissue injury in the brain and that Epha4 is essential to their proinflammatory state.

## Results

### Epha4 is differentially expressed on peripheral immune cells in the whole blood after CCI injury.

**Figure 1:**
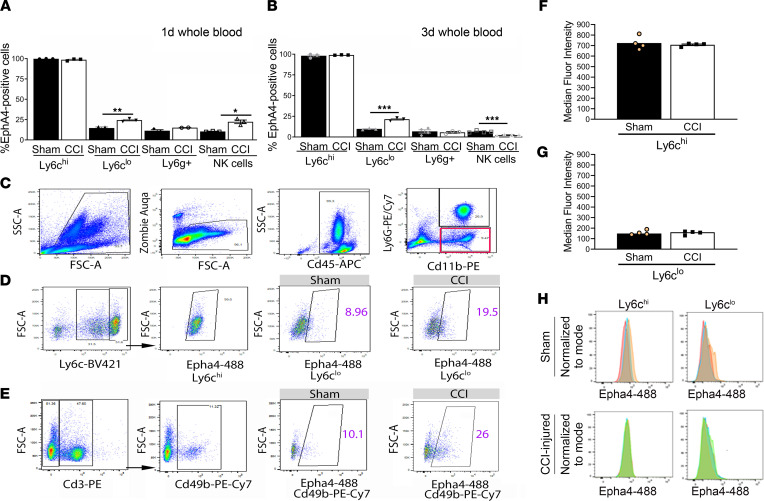
Epha4 is preferentially expressed in peripheral Ly6c^hi^ proinflammatory monocytes in blood after CCI injury. (**A** and **B**) Percentage of Epha4-expressing cells in monocytes (Cd45^+^Cd11b^+^Ly6g^–^Ly6c^hi^ and Cd45^+^Cd11b^+^Ly6g^–^Ly6c^lo^), neutrophils (Cd45^+^Cd11b^+^Ly6g^+^), and NK cells (Cd45^+^Cd3^–^Cd49^+^) (**A**) 1 day after CCI injury and (**B**) 3 days after CCI injury. *n* = 3/group. (**C**) Flow cytometry gating strategy to select (**D**) single, live, Ly6c^hi^, and Ly6c^lo^ monocytes and (**E**) Cd3^–^/Cd49^+^ NK cells expressing Epha4 in whole blood of sham versus CCI-injured mice at 3 dpi. (**F–H**) No change was observed in median fluorescence intensity of Epha4 on Ly6c^hi^ and Ly6c^lo^ cells, respectively, at 3 dpi compared with sham. *n* = 4/group. Statistical analysis was performed using 2-way ANOVA with multiple comparisons test. *P < 0.05, **P < 0.01, ***P < 0.001. Each time point was run separately; data represent 1 independent experiment for each time point.

We found that Epha4 is constitutively expressed on proinflammatory monocytes (CD45^+^CD11b^+^Ly6g^–^Ly6c^hi^) in the whole blood of WT sham and CCI-injured animals (Figure 1, A–C). CCI injury significantly increased the percentage of Epha4 expression in the CD45^+^CD11b^+^Ly6g^–^Ly6c^lo^ monocyte population at 1 (24.4% ± 1.86%) and 3 (21.43% ± 1.05%) days postinjury (dpi) when compared with sham-injured mice at 1 (14.67% ± 1.38%) and 3 dpi (9.77% ± 0.54%) (Figure 1, A–C). This change may influence their inflammatory state under injured conditions. We also show that approximately 10% of circulating neutrophils (CD45^+^CD11b^+^Ly6g^+^) and NK cells (CD45^+^Cd3^–^CD49b^+^) expressed Epha4. While no change was observed in Epha4-expressing neutrophils at 1 and 3 dpi, a transient increase of NK cells was observed at 1 dpi, which was then reduced at 3 dpi (Figure 1, A and B). No changes in Epha4 mean fluorescence intensity were observed in Ly6c cells (Figure 1, D–H). These data demonstrate that Ly6c^hi^ proinflammatory monocytes are enriched for Epha4 in the whole blood.

### BM chimeric Epha4-KO mice show acute neuroprotection and reduced monocyte/macrophage recruitment following CCI injury.

**Figure 2:**
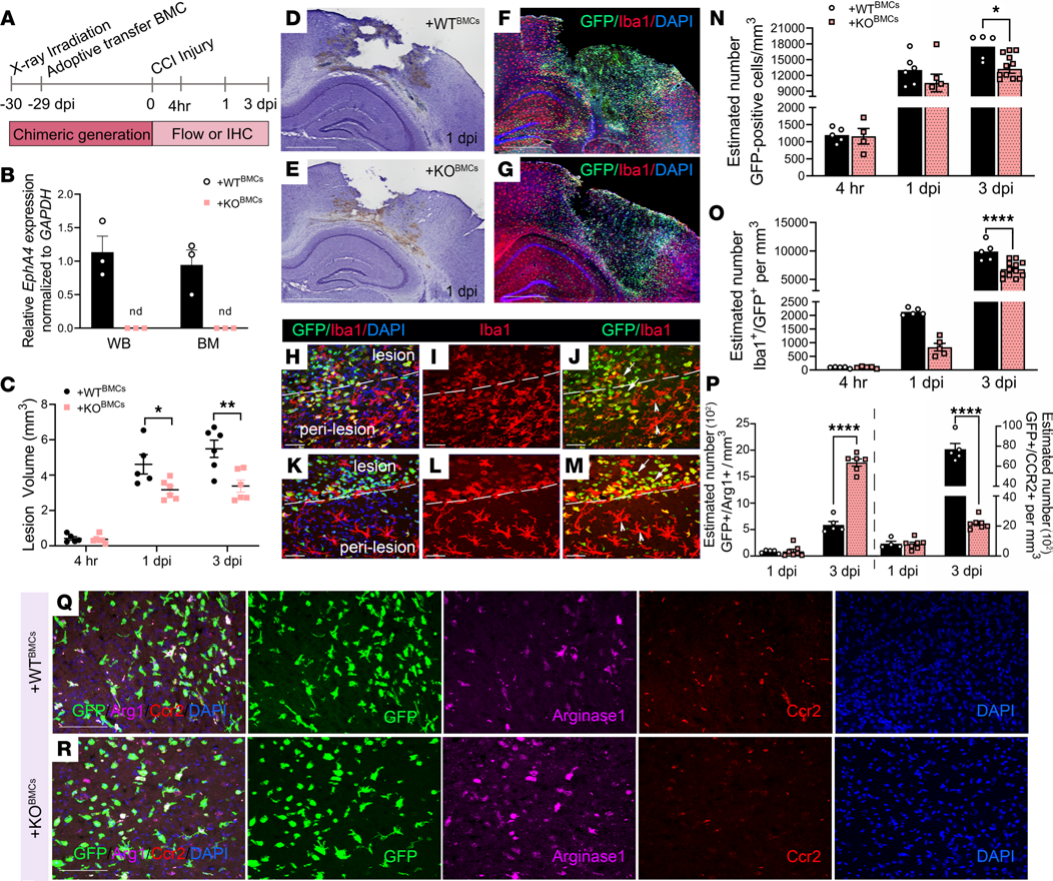
BM chimeric Epha4-KO mice show reduced tissue damage and monocyte/macrophage recruitment following CCI injury. (**A**) Schematic representation of experimental timeline. (**B**) Confirmation of Epha4 deletion in white blood cells, whole blood (WB), and BM cells of Epha4-KO BM chimeric (+KO^BMC^) mice compared with WT (+WT^BMC^) mice. (**C**) Lesion volume (mm^3^) 4 hours, 1 day, and 3 days after injury in +WT^BMC^ and +KO^BMC^ brains using the Cavalieri Estimator. (**D** and **E**) Representative images of Nissl-stained ipsilateral cortex sections from (**D**) +WT^BMC^ and (**E**) +KO^BMC^ at mice 1 dpi. (**F** and **G**) Representative confocal images for Iba1-expressing (red) and infiltrating GFP cells in the perilesion of (**F**) +WT^BMC^ and (**G**) +KO^BMC^ mice at 3 dpi. (**H–M**) Representative images of peripherally derived monocytes/macrophages (Iba1^+^GFP^+^ cells, arrow) and microglia (Iba1^+^GFP^–^ cells, arrowheads) from (**H–J**) +WT^BMC^ and (**K–M**) +KO^BMC^ mice at 3 dpi. (**N** and **O**) Estimated number per mm^3^ of total (**N**) GFP^+^ and (**O**) Iba1^+^/GFP^+^ cells in ipsilateral cortices at 4 hours after infection, 1 dpi, and 3 dpi. (**P**) Estimated number of GFP^+^/Arg^+^ and GFP^+^/Ccr2^+^ cells. (**Q** and **R**) Representative confocal images of GFP/arginase-1/Ccr2/DAPI^+^ cells in the damaged cortex at 3 dpi. Statistical analysis was performed using 2-way ANOVA, repeated measure with multiple comparisons test. *P < 0.05, **P < 0.01, ****P < 0.0001. *n* = 5–12 mice per group. Combined data are from multiple independent experiments. Scale bar: 1 mm (**D–G**); 100 μm (**Q** and **R**); 50 μm (**H–M**). nd, not detected.

To assess the role of peripheral immune-derived Epha4 on TBI outcome, we generated GFP BM chimeric mice that were deficient in Epha4 in BM-derived cells (BMCs) from *Epha4^fl/fl^/Rosa^mTmG^/Tek-Cre* or *Epha4^+/+^/Rosa^mTmG^/Tek-Cre* mice and as we previously described (14). We found that the transcript for Epha4 was undetected in the whole blood and BM of *Epha4*-BM–deficient mice (+KO^BMC^) mice compared with WT (+WT^BMC^) mice (Figure 2B). These mice were subjected to the CCI injury model following reconstitution protocol (Figure 2A). We quantified the lesion volume (mm^3^) on serial coronal sections at 4 hours and 1 and 3 dpi using Cavalieri probe, as performed previously (14, 18, 19). We found a significant difference at 1 (3.17 ± 0.23 mm^3^) and 3 dpi (3.38 ± 0.34 mm^3^; *P* = 0.01) in +KO^BMC^ compared with +WT^BMC^ mice (4.61 ± 0.55 mm^3^ and 5.49 ± 0.49 mm^3^, respectively) (Figure 2, C–E). We also found that the +KO^BMC^ mice showed a significant reduction in the total number of GFP^+^ cells that infiltrate the damaged tissue at 3 dpi (13,534 ± 975 mm^3^; *P* = 0.04) compared with +WT^BMC^ (17,473 ± 1069 mm^3^) (Figure 2, F, G, and N). In addition, the estimated number of GFP^+^/Iba1^+^ monocytes/macrophages was also reduced in +KO^BMC^ mice at 1 dpi (918 ± 131 cells/mm^3^; *P* = 0.0001) compared with that in +WT^BMC^ mice (2132 ± 57 cells/mm^3^) and at 3 dpi (6795.9 ± 384.8 vs. 9903.8 ± 740.3 cells/mm^3^; respectively) (Figure 2, H–M and O). Moreover, the number of proinflammatory Ccr2^+^ cells was significantly increased (7702.82 ± 516.75 cells/mm^3^; *P* = 0.0001) on +WT^BMC^ cells (Figure 2, P and Q; right *y* axis) compared with +KO^BMC^ GFP^+^ cells (2259.4 ± 211.28 cells/mm^3^) at 3 dpi (Figure 2, P and R; right *y* axis). Conversely, the number of antiinflammatory, arginase-1^+^ macrophages was significantly increased (1653.5 ± 118.97 cells/mm^3^; *P* = 0.0001) in +KO^BMC^ mice compared with +WT^BMC^ mice (588.4 ± 58.75 cells/mm^3^) (Figure 2, P–R; left *y* axis).

In addition, +WT^BMC^ mice subjected to CCI injury showed significant motor deficits at 3 dpi (51.06% vs. baseline; *P* < 0.001), 7 dpi (69.46% vs. baseline; *P* < 0.01), and 14 dpi (80.22% vs. baseline; *P* < 0.05) compared with sham mice, while +KO^BMC^ mice did not shown statistical difference (89.9% at 3 dpi; 96.96% at 7 dpi; and 113.9% at 14 dpi vs. baseline, respectively) in motor impairment compared with sham mice (Supplemental Figure 1; supplemental material available online with this article; https://doi.org/10.1172/jci.insight.156319DS1). These data suggest that peripherally derived immune cell recruitment and polarization state may be tightly regulated by Epha4, which regulates functional outcome.

### Epha4 deficiency modifies phenotypic gene expression on peripherally derived monocytes/macrophages.

**Figure 3:**
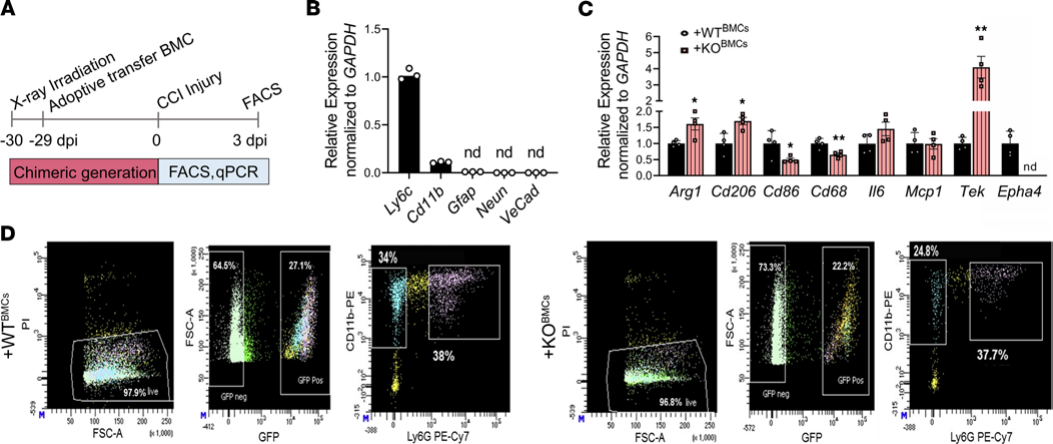
Peripheral deficiency of Epha4 shifts the monocyte/macrophage population in the brain toward the antiinflammatory phenotype after CCI injury. (**A**) Schematic representation of the experimental timeline. FACS was performed to isolate GFP^+^CD11bLy6g^–^ monocytes/macrophages from ipsilateral cortices at 3 dpi. (**B**) The purity was tested using qRT-PCR analysis of *Ly6c*, *CD11b*, *Gfap*, *NeuN*, and *VeCadherin* (*VeCad*). (**C**) Relative mRNA expression of antiinflammatory markers *Arg1*and *Cd206;* proinflammatory markers *Cd86, Cd68*, *Ccl2,* and *Il6*; provascular repair receptor *Tek* (*Tie2*); and Epha4 was measured in monocytes isolated from +WT^BMC^ and +KO^BMC^ mice using qRT-PCR. (**D**) Flow cytometry gating strategy to select live, GFP^+^, CD11b^+^, and Ly6g^–^ monocytes from +WT^BMC^ and +KO^BMC^ mice. Statistical analysis was performed using Student’s *t* test. *P < 0.05, **P < 0.01, ****P < 0.0001. *n* = 5 mice per group. Combined data are from multiple independent experiments. nd, not detected.

In order to investigate the pro- or antiinflammatory phenotype of infiltrating monocytes, we utilized flow cytometry to sort GFP^+^CD11b^+^Ly6g^–^ monocytes from the ipsilateral cortices at 3 dpi of +WT^BMC^ and +KO^BMC^ mice (Figure 3, A and D). RNA isolated from flow-sorted cells showed high expression levels of *Cd11b* and *Ly6c* (monocytes), but no transcript was observed for *Gfap* (astrocytes), *Rbfox3 (NeuN*, neurons), and *Ve-cadherin* (*Cdh5*, endothelial cells) relative to *Ly6c* (Figure 3B). Sorted KO cells lacked mRNA transcripts for Epha4 but displayed increased arginase-1 (*Arg1*) and *Mrc1* (*Cd206*), prominent markers for antiinflammatory M2-like monocytes/macrophages, and decreased proinflammatory *Cd68* and *Cd86* (Figure 3C). No change was observed in *Il6* or *Ccl2* expression. Interestingly, monocytes derived from +KO^BMC^ mice expressed high levels of *Tek* (*Tie2)* transcripts compared with +WT^BMC^ mice. These data suggest that, in addition to reduced recruitment of monocytes/macrophages in *Epha4*-BM–deficient mice, the polarization state of monocytes/macrophages may be skewed toward an antiinflammatory state in damaged neural tissue.

### Monocyte suppression reduces their presence and proinflammatory phenotype in the damaged tissue.

**Figure 4:**
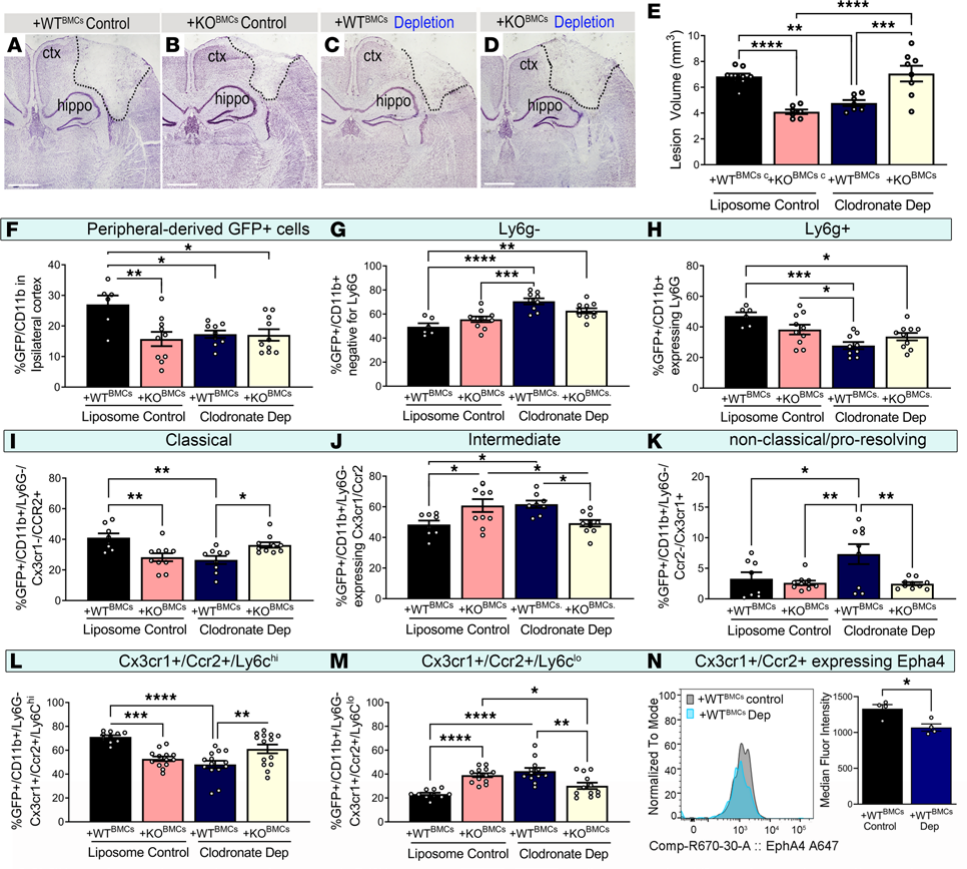
Monocyte depletion and loss of Epha4 alters tissue damage, peripheral monocyte influx, and phenotype after brain injury. (**A–D**) Representative images of the ipsilateral cortices of Nissl-stained coronal sections from (**A**) liposome control–treated +WT^BMC^ (nondepleted), (**B**) liposome control–treated +KO^BMC^ (nondepleted), (**C**) clodronate-treated +WT^BMC^ (monocyte depletion), and (**D**) clodronate-treated +KO^BMC^ (monocyte depletion) mice at 3 dpi. Tile image magnification x4. (**E**) Lesion volume (mm^3^) at 3 dpi was estimated in 5 Nissl-stained serial coronal sections using the Cavalieri Estimator. (**F–N**) Flow cytometry analysis was used to examine the differential abundance of peripheral GFP^+^ immune cell populations infiltrating the ipsilateral cortices of liposome control–treated +WT^BMC^, liposome control–treated +KO^BMC^, clodronate-treated +WT^BMC^, and clodronate-treated +KO^BMC^ mice. Using FACS, we found the percentage of peripherally derived (**F**) GFP^+^CD11b^+^ cells, (**G**) monocytes (GFP^+^CD11b^+^Ly6g^–^), (**H**) neutrophils (GFP^+^CD11b^+^Ly6g^+^), (**I**) classical monocytes (GFP^+^CD11b^+^Ly6g^–^Cx3cr1^–^Ccr2^+^), (**J**) intermediate monocytes (GFP^+^CD11b^+^Ly6g^–^Cx3cr1^+^Ccr2^+^), and (**K**) nonclassical monocytes (GFP^+^CD11b^+^Ly6g^–^Ccr2^–^Cx3cr1^+^). FACS also showed changes in (**L**) intermediate Ly6c^hi^ (GFP^+^CD11b^+^Ly6g^–^Cx3cr1^+^Ccr2^+^Ly6c^hi^) and (**M**) Ly6c^lo^ (GFP^+^CD11b^+^Ly6g^–^Cx3cr1^+^Ccr2^+^Ly6c^lo^) monocytes. (**N**) Epha4 mean fluorescence intensity on intermediate monocytes (GFP^+^CD11b^+^Ly6g^–^Cx3cr1^+^Ccr2^+^Epha4) from control- and clodronate-treated +WT^BMC^ mice is reduced in depleted WT mice. Statistical analysis was performed using 1-way ANOVA with multiple comparisons test. *P < 0.05, **P < 0.01, ***P < 0.001, ****P < 0.0001. *n* = 6–8 mice per group. Combined data are from multiple independent experiments. Ctx, cortex; hippo, hippocampus; Dep, depleted.

To determine whether reducing the presence of peripherally derived monocytes/macrophages alone may contribute to neuroprotection, we performed monocyte depletion prior to CCI injury in BM chimeric mice using clodronate liposomes. This treatment resulted in an approximately 80% reduction in circulating monocytes, with no effect on neutrophils and lymphocytes (Supplemental Figure 2, A–D). We found that control-treated +KO^BMC^ mice showed reduced lesion volume (4.10 ± 0.18 mm^3^; *P* = 0.0001) compared with that of control +WT^BMC^ mice (6.84 ± 0.22 mm^3^) (Figure 4, A, B, and E). Monocyte suppression substantially reduced the lesion volume in +WT^BMC^ mice (4.78 ± 0.23 mm^3^; *P* = 0.002) (Figure 4, C and E). This suggests that suppression of peripherally derived monocytes may ameliorate tissue damage, as previously shown (2). However, the effects of clodronate surprisingly attenuated tissue protection +KO^BMC^ chimeric mice (7.06 ± 0.61 mm^3^) (Figure 4D), even though cell recruitment was reduced (Figure 4F). This suggest that total influx of immune cells is dispensable in neural tissue damage.

Next, we utilized flow cytometry to examine the percentage of the different GFP^+^ cell populations in the neural dissociated, microdissected 4 × 4 mm piece of ipsilateral cortex with and without monocyte depletion (Supplemental Figure 1F; gating strategy). First, we observed that the percentage of GFP/CD11b^+^ cells was significantly reduced in liposome control–treated +KO^BMC^, clodronate-treated +WT^BMC^ (with depleted monocytes), and clodronate-treated +KO^BMC^ (with depleted monocytes) mice compared with liposome control–treated +WT^BMC^ mice (Figure 4F), indicating an overall reduction in cell recruitment at 3 dpi. The GFP^+^ cell reduction in the control +KO^BMC^ mice, compared with control +WT^BMC^ mice, confirmed the stereology findings in Figure 2N. When evaluating the GFP^+^CD11b^+^ cell population, we observed that cells isolated from control +KO^BMC^ mice showed no change in the percentage of Ly6g^–^ cells (monocytes, dendritic cells, NK cells) or Ly6g^+^ cells (neutrophils) compared with +WT^BMC^ mice (Figure 4, G and H, respectively). However, the percentage of the GFP^+^CD11b^+^Ly6g^–^ cell population that was Cx3cr1^–^Ccr2^+^ (classical monocytes) was significantly reduced, whereas the Cx3cr1^+^Ccr2^+^ population (intermediate monocytes) was significantly increased in control +KO^BMC^ mice when compared with control +WT^BMC^ mice (Figure 4, I and J; respectively). Moreover, the intermediate Cx3cr1^+^Ccr2^+^ population had a reduced percentage of Ly6c^hi^ cells (proinflammatory) and increased percentage of Ly6c^lo^ cells (antiinflammatory) in control +KO^BMC^ mice (Figure 4, L and M, respectively). When evaluating the Cx3cr1/Ccr2 double-positive intermediate cells from +WT^BMC^ mice treated with clodronate, we found reduced intensity of Epha4 by flow cytometry compared with controls (Figure 4N). This suggests that Epha4 regulates total cell recruitment and phenotypic identity of infiltrating monocytes.

To test this, we evaluated the effects of clodronate liposome treatment in +WT^BMC^ mice. Surprisingly, peripheral monocyte depletion resulted in a significant increase in the percentage of GFP/CD11b/Ly6g^–^ cells (Figure 4G), concomitant with a decrease in the percentage of GFP/CD11b/Ly6g^+^ neutrophils (Figure 4H). Furthermore, the Ly6g^–^ population had a reduced percentage of Cx3cr1^–^Ccr2^+^ (classical) along with an increased percentage of both Cx3cr1^+^Ccr2^+^ (intermediate) and Cx3cr1^+^Ccr2^–^ (nonclassical) cell populations (Figure 4, I–K; respectively). Moreover, the intermediate Cx3cr1^+^Ccr2^+^ cells showed a reduced percentage of Ly6c^hi^ cells (proinflammatory monocytes) and an increased percentage of Ly6c^lo^ cells (antiinflammatory) in clodronate liposome–treated +WT^BMC^ mice (Figure 4, L and M). Importantly, the Cx3cr1/Ccr2 double-positive Ly6g^–^ population showed reduced median fluorescence intensity of Epha4 expression in clodronate-treated +WT^BMC^ mice when compared with liposome control–treated mice (Figure 4N) which is correlated with the neuroprotection observed in the same group (Figure 4E).

Interestingly, clodronate liposome–treated +KO^BMC^ mice also displayed reduced GFP^+^CD11b^+^ immune cell recruitment compared with liposome control–treated +WT^BMC^ mice (Figure 4F). However, an increase in the Cx3cr1/Ccr2 double-positive Ly6g^–^ population was not observed compared with WT mice (Figure 4J), which correlated with attenuation of neuroprotection (Figure 4, D and E). Importantly, an increase in the percentage of the Cx3cr1/Ccr2 double-positive Ly6g^–^ population (Figure 4J) correlated with neuroprotection and was prevented in clodronate liposome–treated +KO^BMC^ mice. Taken together, these data suggest that, while Epha4 mediates recruitment and polarization state of monocytes, their phenotypic state, not overall influx, contributes to tissue damage.

Indeed, cultured Epha4-null monocytes/macrophages displayed reduced proinflammatory *Ccl2* and *Il6*, along with increased proresolving marker *Arg1* and antiinflammatory *Il10* and *Il4* cytokine mRNA expression, compared with WT cells following LPS stimulation (Supplemental Figure 3). These studies demonstrate that Epha4 mediates the proinflammatory phenotype of monocytes/macrophages under inflammatory conditions.

### Tie2 receptor expression correlates with the antiinflammatory polarization phenotype in the CCI-injured cortex.

**Figure 5:**
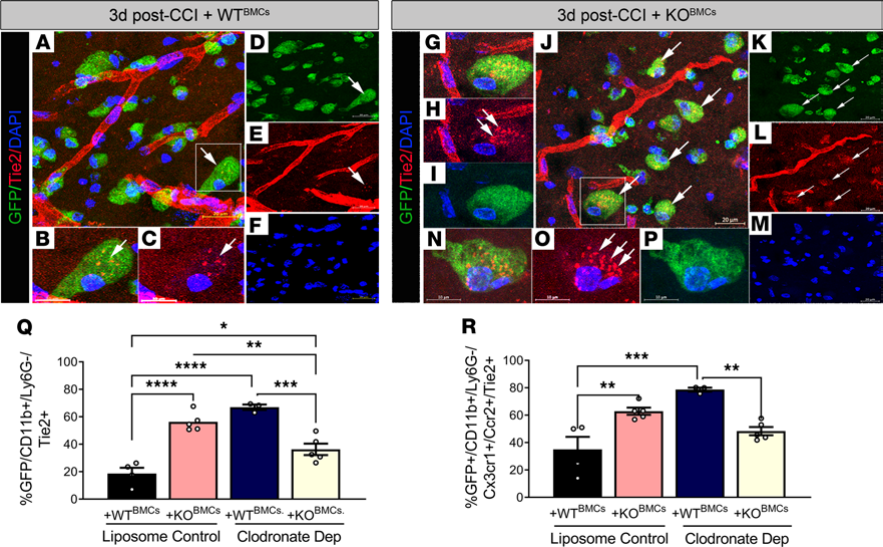
Peripheral Epha4 deficiency and monocyte depletion increase Tie2 receptor expression on Cx3cr1^+^/cr2^+^ cells in the CCI-injured cortex. Representative confocal images showing Tie2 receptor expression (red) in infiltrating GFP^+^ immune cells (arrows) at the perilesion of the ipsilateral cortices of (**A–F**) +WT^BMC^ and (**G–P**) +KO^BMC^ mice 3 days after CCI injury. (**Q**) Flow cytometry analysis was used to examine the percentage of Tie2-expressing cells among GFP^+^CD11b^+^Ly6g^–^ cells and (**R**) GFP^+^CD11b^+^Ly6g^–^Cx3cr1^+^Ccr2^+^ cell populations in the ipsilateral cortices of liposome control–treated +WT^BMC^, liposome control–treated +KO^BMC^, clodronate-treated +WT^BMC^, and clodronate-treated +KO^BMC^ mice. Statistical analysis was performed using 2-way ANOVA with multiple comparisons test. *n* = 3–5/group. *P < 0.05, **P < 0.01, ***P < 0.001, ****P < 0.0001. Combined data are from multiple independent experiments. Scale bar: 20 μm (**A**, **J**, and **K–M**); 10 μm (**B**, **C**, and **N–P**). The boxed region in **A** is shown at higher magnification in **B** and **C**, and the boxed region in **J** is shown at higher magnification in **G–I**. Original magnification: x40.

We previously demonstrated that cultured Epha4-null monocytes/macrophages display a significant increase in Tek mRNA expression (14); we also show that flow-sorted infiltrating GFP/CD11b/Ly6g^–^ cells from +KO^BMC^ mice displayed higher Tek transcript levels (Figure 3C) when compared with +WT^BMC^ mice. Tie2-expressing monocytes/macrophages are well studied in tumor angiogenesis (20) and are gaining interest for their possible role in CNS repair (21–23). Using immunohistochemistry and confocal image analysis, we observed greater numbers of GFP^+^ cells that colabeled with Tie2 in the CCI-injured perilesional cortices of +KO^BMC^ mice (Figure 5, G–P) compared with +WT^BMC^ mice (Figure 5, A–F) at 3 dpi. Interestingly, we found that Tie2 expression in +KO^BMC^ cells was contained in larger, more numerous clusters (Figure 5, N–P), which may implicate the receptor activation (24).

Next, using flow cytometry, we sought to investigate whether Tie2 expression on GFP/CD11b/Ly6g^–^ cells infiltrating the injured cortex correlated with the phenotypic shift induced by clodronate liposome and Epha4 loss of function in BMCs. Similar to our histological findings, we observed a significant increase in the percentage of GFP^+^CD11b^+^Ly6g^–^ cells that expressed Tie2 of approximately 30% in control liposome–treated +KO^BMC^ mice and approximately 40% in clodronate liposome–treated +WT^BMC^ mice compared with +WT^BMC^ controls (Figure 5Q and Supplemental Figure 4). The Cx3cr1/Ccr2 double-positive Ly6g^–^ population had the greatest increase in Tie2 expression under these conditions (Figure 5R). Paradoxically, clodronate liposome–treated +KO^BMC^ mice had attenuated expression of Tie2 on these cell populations, concomitant with a loss of neuroprotection and monocyte phenotypic shift (Figure 5, Q and R). These data suggest that the expression of Tie2 receptor on the Ly6g^–^Cx3cr1^+^Ccr2^+^ cell population may influence monocyte/macrophage activation state and tissue protection.

### Tie2 regulates the antiinflammatory state of Epha4-null monocytes/macrophages.

**Figure 6:**
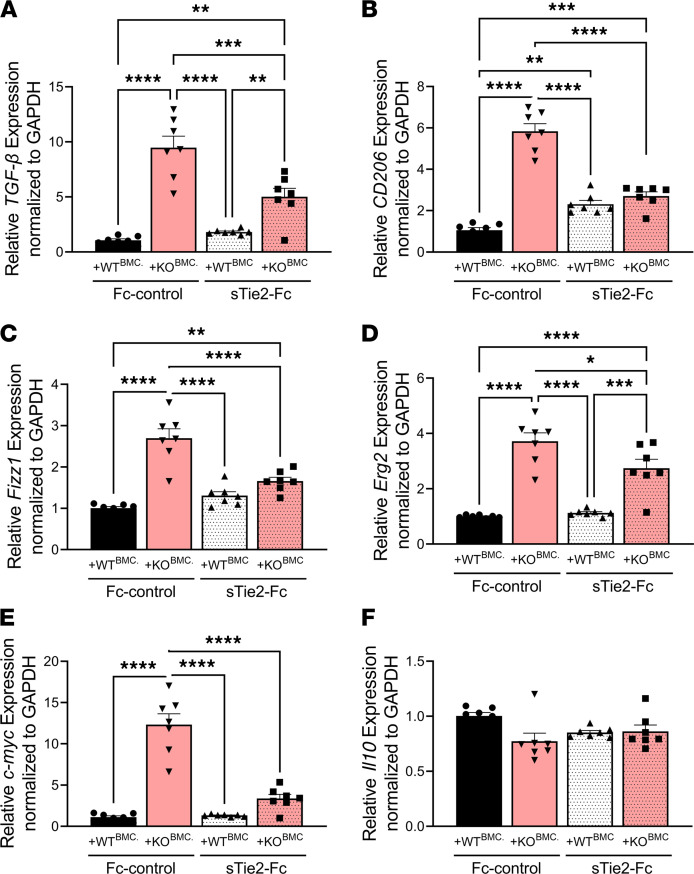
Cultured Epha4-null monocytes/macrophages show elevated M2-like gene expression that is attenuated following Tie2 receptor blockade. BM-derived monocytes/macrophages (BMCs) were isolated from WT and Epha4-KO mice and cultured in 10 ng/mL M-CSF for 5 days. Cells were then treated with 20 ng/mL IL-4 for 48 hours, followed by 20 μg/mL soluble recombinant mouse Tie2-Fc (sTie2-Fc) or Fc-control for 5 hours. The relative expression of (**A**) *TGF-β*, (**B**) *CD206*, (**C**) *Fizz1*, (**D**) *Erg2*, and (**E**) *c-myc* was significantly increased in BM-derived monocytes/macrophages from KO compared with WT cells. (**F**) No difference was observed in *Il10* expression. Blockade of Tie2 signaling by sTie2 attenuated the M2-like genes in KO cells. Six independent wells per group were used for in vitro assay. Statistical analysis was performed using 1-way ANOVA with multiple comparisons test. *P < 0.05, **P < 0.01, ***P < 0.001, ****P < 0.0001. Expression was normalized to *GAPDH* and then analyzed and graphed relative to the WT Fc-control group.

Our previous findings demonstrate increased expression of *Tek* (*Tie2*) and *Arg1*, a M2-like macrophage marker (25), in Epha4-KO alternatively activated macrophages (14) and in monocytes derived from the injured cortices of +KO^BMC^ mice (Figure 3C). To determine whether Tie2 mediates the antiinflammatory phenotypic activation of Epha4-null monocytes/macrophages, we inhibited Tie2 signaling using 20 μg/mL soluble ectodomain Tie2-Fc (sTie2-Fc) in M2-polarized BMCs isolated from WT and KO mice. We found that, following exposure to Fc-control, a significant increase in the antiinflammatory M2-like genes *TGF-β* (Figure 6A)*, CD206* (Figure 6B)*, Fizz1* (Figure 6C)*, Erg2* (Figure 6D), and *c-myc* (Figure 6E), but not *Il10* (Figure 6F), was observed in KO compared with WT cells. Importantly, inhibition of Tie2 signaling using sTie2-Fc attenuated the expression of these key genes involved in M2-like phenotypic activation of Epha4-null monocytes/macrophages. This demonstrates Epha4 limits the antiinflammatory state of monocytes/macrophages by restricting Tie2 signaling.

## Discussion

Eph receptor signaling has emerged as a central player in the regulation of CNS development and injury (26). Using a BM chimeric approach, we found that peripheral immune cell deficiency of Epha4, which is highly expressed on Ly6c^hi^ monocytes, is neuroprotective. Epha4-null monocytes isolated from the ipsilateral cortex showed a significant increase in antiinflammatory gene expression and a prominent shift in their surface markers from classically activated toward an intermediately activated subset, which expressed both proinflammatory Ccr2 and proresolving Cx3cr1 markers. Strikingly, this subpopulation found in the ipsilateral cortices of +KO^BMC^ mice displays increased proportions of antiinflammatory, Ly6c^lo^-expressing, and reduced proinflammatory Ly6c^hi^-expressing cells. This population also shows increased Tek (Tie2) cell percentages compared with WT controls. Moreover, a similar neuroprotective response, including an antiinflammatory monocyte/macrophage phenotypic shift was observed in WT mice treated with clodronate. Our findings demonstrate that a member of the Eph receptor tyrosine kinase family, Epha4, mediated monocyte/macrophage infiltration and polarization state in the brain as a consequence of trauma.

Previous studies revealed that patients with TBI show an elevation in absolute monocyte count in blood within 24 hours of injury (27). Within days, monocytes infiltrate the perivascular spaces and brain parenchyma, and they can reside there for weeks (28). Cytokines produced at the site of injury activate peripherally derived macrophages into classical, alternative (nonclassical), or intermediate phenotypes. Clinical and preclinical studies reported the upregulation of both proinflammatory (IL-1b, IL-18, IL-6, and TNF) and antiinflammatory (IL-10 and TGF-β) cytokines, explaining the existence of multiple macrophage phenotypes in the injured brain (29). Given that the classical (proinflammatory) subtype exacerbates brain injury while the alternative (antiinflammatory) subtype promotes wound healing and dead cell clearance (30), we postulated that modulation of macrophage polarization may represent a key process in controlling secondary damage following injury.

It has been established that monocytes/macrophages in mice are pro- and antiinflammatory, based on Ly6c^hi^ and Ly6c^lo^ expression, respectively. In humans, CD14^hi^CD16^−^ and CD14^lo^CD16^+^ cell surface proteins identify and distinguish the 2 major pro- and antiinflammatory monocyte subsets, respectively (31). Though differences exist, both mouse Ly6c^hi^ and human CD14^hi^CD16^−^ cells highly express chemokine receptor Ccr2; while Ly6c^lo^ and CD14^lo^CD16^+^ cells have differential expression, with elevated levels of fractalkine receptor Cx3cr1. These divergent populations also exhibit many analogous expression profiles (32). In the current study, we used proinflammatory (Ccr2 and Ly6c) and antiinflammatory (Cx3cr1) surface markers to identify monocyte/macrophage populations. We found that 70% of these colabeled cells were Ly6c^hi^, suggesting the proinflammatory nature of the majority of monocytes that infiltrate the brain after injury.

Although neuroinflammation is now recognized as a major contributor to injury outcome, the use of wide-ranging antiinflammatory and immunosuppressive agents has failed to improve TBI outcome in human clinical trials (33). These agents cause general immune dampening, which may interfere with both proinflammatory as well as important proresolving functions of monocytes/macrophages (30). However, the results of our study comport with previous findings that show that global monocyte depletion using clodronate liposome ameliorates TBI-induced neutrophil and monocyte infiltration, brain edema, tissue damage, motor deficit, and memory impairment (2, 34). The neuroprotective effects of clodronate may be attributed to the reduction of immune cell infiltration. However, our findings uncover a surprising shift in subtype polarization in clodronate-pretreated mice subjected to CCI injury. The classical proinflammatory (Ccr2^+^Cx3cr1^–^) population was replaced with intermediately activated (Ccr2^+^Cx3cr1^+^) monocytes/macrophages that showed high expression of Tie2 and reduced expression Epha4, correlated with tissue protection. The flow-sorted immune population further demonstrated increased mRNA levels of antiinflammatory markers Arg1 and CD206. These observations demonstrate that the modulation of monocyte/macrophage phenotypic state, and not overall recruitment, dictates outcome, suggesting that a therapeutic approach for retooling or controlling monocyte/macrophage phenotypic fate may be a viable strategy for treating brain injury.

This study further showed that clodronate treatment attenuates the neuroprotection observed in +KO^BMC^ chimeric mice, even though the overall recruitment of cells was reduced, similar to that in clodronate-treated +WT^BMC^ mice. This correlated with attenuation of the antiinflammatory polarization shift of infiltrating monocytes/macrophages. While the mechanisms of this response to clodronate treatment in +KO^BMC^ mice remain unclear, it is possible that additional compensation from the BM may contribute to the observed phenotype, which requires further investigation. Moreover, because clodronate may also affect dendritic cells, we cannot rule out the possibility of their contribution. Taken together, this study purports that drug depletion of monocytes not only reduces the infiltration of peripherally derived immune cells, but has a dynamic effect on their polarization shift from proinflammatory to antiinflammatory in an animal model of brain injury that was an unexpected. Attenuation of their polarization, and not cell influx, prevented neuroprotection, confirming that the phenotypic state of monocytes/macrophages dictates brain injury–induced damage.

Tie2 receptor tyrosine kinase is expressed on endothelial cells and monocytes/macrophages, which regulate differentiation, survival, hematopoiesis, and adhesion (35). Tie2 is postulated to skew monocytes/macrophages toward antiinflammatory polarization to promote angiogenesis (36). Our findings suggest an inverse relationship between Epha4 expression and Tie2 activation on monocytes/macrophages. In the absence of Epha4, Tie2 is upregulated and clustered on peripherally derived cells in the ipsilateral cortex following CCI injury. Moreover, our in vitro experiments revealed that Epha4 suppresses the M2-like phenotypic state in alternatively activated monocytes/macrophages by limiting Tie2 signaling. This is consistent with our previous findings that show Epha4-deficient M2 monocytes/macrophages display increased expression of *Tek* (14). Taken together, these data suggest that Epha4/Tie2 coordination controls peripherally derived innate immune regulation in the murine brain after TBI.

In summary, peripheral monocytes are an essential component of the innate immune system; they act as a double-edged sword in the cortical neuroinflammatory milieu. Here, we identified an intermediate monocyte subtype, with an antiinflammatory state that is negatively regulated by a member of the Eph receptor tyrosine kinases in the injured brain. Regulation of monocyte/macrophage polarization remains under intense investigation. This study provides important mechanistic insight regarding the key drivers of the phenotypic state of monocytes/macrophages in the brain following trauma.

## Methods

### Animals.

All mice were housed in an AAALAC-accredited, virus/specific antigen–free facility with a 12-hour light/dark cycle and had access to food and water ad libitum. All mice used in these studies were male mice in order to reduce variables with sex differences. CD1 mice were purchased from Charles Rivers and reared until age P60–P90 for experimentation. *Epha4*^fl/fl^ recipient as well as *Epha4^+/+^/ROSA^mTmG^/Tek-Cre* and *Epha4^fl/fl^/ROSA^mTmG^/Tek-Cre* donor mice used for chimeric generation were on the CD1 background (at least 10 generations) and bred; genotyping was as previously described (14, 17, 37).

### Adoptive transfer.

BM chimeric mice were generated as previously described (14). *Epha4^fl/fl^* male mice were x-ray irradiated without head shield. Mice were given 1 mg/mL gentamycin sulfate water for 3 days prior and 2 weeks following irradiation. Within 24 hours of irradiation, recipient mice received 3 million BMCs from donors via tail vein injection. BMCs were flushed from the femurs and tibias of donor male mice (Rosa26*^mtmg^* /*Tek-Cre/Epha4^+/+^ or* Rosa26 *^mtmg^*/*Tek-Cre/Epha4^fl/fl^*) into FBS-containing DMEM media.

### CCI.

CCI injury was performed as previously described (14). Briefly, adult male mice were anesthetized and positioned in a stereotaxic frame. A 4 mm craniotomy was made over the right parietal-temporal cortex using a portable drill. CCI injury was induced using the eCCI-6.3 device (Custom Design & Fabrication LLC) connected to 3 mm rounded tip at a velocity of 5.0 m/s, depth of 2.0 mm, and duration of 100 milliseconds. After injury, mice were monitored until fully recovered from anesthesia.

### Rotarod.

All mice were habituated to the experimental room for 1 hour prior to testing. Equipment was cleaned with 70% ethanol before and after evaluation of each mouse. A rotarod (Economex; Columbus Instruments) was used to assess gross motor function as previously described (18, 19). Animals were trained on the rotarod from day –4 to day –2, and a baseline measurement was taken on day –1. On day 0, either CCI or sham injury was performed. Measurements were then taken at 3 days, 7 days, and 14 days after CCI or after sham injury. For each recording session, mice were placed on the rotarod, and the rotarod was allowed to rotate with a starting rotational speed of 10 rpm and acceleration of 0.1 rpm/s. The time to fall was recorded for each mouse, and the mean time to fall of individual mice at 3, 7, and 14 days was reported as relative to the baseline for data analysis.

### Evaluation of lesion volume and cell counts.

Lesion volume (mm^3^) and cell counts were measured blindly as previously described using a StereoInvestigator (MicroBrightField) connected to an upright Olympus BX51TRF motorized microscope (Olympus America) (14). Briefly, 30 μm serial coronal sections (5 sections with interval of 15 sections, at −1.1 to −2.6 mm posterior from bregma) were stained with Nissl, and Cavalieri Estimator (StereoInvestigator) was used to measure the volume of contusion based on section thickness, section interval, and total number of sections within the Cavalieri probe. Cell counts were analyzed in the entire dorsal cortex contour, using the optical fractionator with a grid size set at 450 × 450 mm and 150 × 150 mm counting frame.

### Immunohistochemistry and confocal image analysis.

Mice were anesthetized using isoflurane overdose and then perfused with 20 mL ice-cold 1× PBS, followed by 50 mL 4%PFA. Brains were postfixed overnight in PFA and then cryopreserved and sectioned as previously described (14). The 30 μm serial sections were blocked in 2% cold water fish gelatin (MilliporeSigma.) in 0.2% Triton for 1 hour and then incubated with Gt Anti-Tie2 (R&D Systems) Ab (1:200), Rb anti-Iba1 (Wako) Ab (1:200), Rb anti-Ccr2 (Abcam) Ab (1:200), and/or Gt anti-arginase-1 (1:500) in block overnight; washed with 1× PBS; and then treated with appropriate secondary Abs (Invitrogen): Alexa Fluor donkey anti-rabbit 594, Alexa Fluor donkey anti-rabbit 647, Alexa Fluor donkey anti-goat 594, and Alexa Fluor donkey anti-goat 647 (1:250 in block) for 1 hour. The sections were washed and mounted in media with DAPI counterstain (SouthernBiotech). Images were acquired using a Zeiss 880 confocal microscope (Carl-Zeiss).

### Quantitative real-time PCR.

Total RNA from macrophage culture was isolated according to the manufacturer’s instructions using TRIzol reagent (Ambion), and total RNA was isolated from the blood using TRIzol Reagent LS (ThermoFisher) per the manufacturer’s instructions. RNA quantification was carried out by measuring absorbance with spectrophotometer ND-1000 (NanoDrop). Samples were DNAse treated using a DNAse1 amplification grade kit (MilliporeSigma). RNA was reverse transcribed into cDNA with an iScript cDNA synthesis kit (Bio-Rad) per the manufacturer’s specifications. For quantitative real-time PCR (qRT-PCR) analysis, 50 ng cDNA per reaction was amplified using iTaq Universal SYBR Green Supermix (Bio-Rad). Expression changes were calculated using ΔCq values with reference to *β-actin* internal control for cultured cells and *Gapdh* for all other samples. Relative expression was calculated and then normalized to appropriate sham or untreated samples and represented as fold change. All primers (*Arg1*, *Cd206*, *Cd86*, *Il6*, *Mcp1*, *Tek*, *Epha4*, *Tgfb*, *Fizz1*, *Erg2*, *m-myc*, and *Il10*) were tested for primer efficiency, which ranged from 90% to 110%.

### Cell culture.

BMCs isolated from 8- to 10-week-old Epha4-WT (*Rosa26^mtmg^/Tek-Cre/Epha4^+/+^*) or Epha4-KO (*Rosa26^mtmg^/Tek-Cre/Epha4^fl/fl^*) mice were cultured in complete RPMI medium supplemented with 10% fetal bovine serum, 2 mM L-glutamine, 1% penicillin/streptomycin, and 10 ng/mL M-CSF. Briefly, BMCs were isolated from the femurs and filtered through a 70 μm filter; red blood cells were lysed using ACK lysing buffer (Gibco); and cells were cultured at 1 × 10^6^ cells/mL in complete RPMI medium. Cells received fresh complete media containing 10 ng/mL M-CSF on days 2 and 4. On day 5, BMCs were allowed to equilibrate for 2 hours in fresh RPMI medium prior to treatment with either 1 μg/mL 

### E.

*coli* O111:B4 LPS (MilliporeSigma) for 4 hours or 20 ng/mL IL-4 (R&D Systems) for 48 hours followed by 20 μg/mL recombinant mouse sTie2-Fc or Fc-control (Sino Biological) for 5 hours.

### Monocyte depletion.

Male mice (25–35 g) were placed on antibiotic water (1 mg/mL Gentamicin) 3 days prior to first clodronate or PBS-encapsulated liposome injection (Liposoma BV). All injections were performed via tail vein. First, liposome injection occurred 4 days prior to CCI injury (200 μL at 5 mg/mL), and then a second injection was performed 1 day prior to CCI injury.

### Flow cytometry.

Aliquots of approximately 10^6^ cells/100 μL in 1× PBS (Fisher Scientific) were put into 96-well round-bottom microtiter plates. To block nonspecific binding, the cells were first incubated with anti-CD16/32 mAb (BioLegend) for 15 minutes on ice and then appropriate conjugated or unconjugated primary mAbs (BioLegend, CD11b, Ly6c, Ccr2, Ly6g, CD202b (Tek), Ptprc (CD45), Cx3cr1, CD49b, Cd3, CD115) and Epha4 (Proteintech) and incubated for 20 minutes. Controls included cells incubated with or without fluorochrome-conjugated control Abs and with unspecific isotype Ab, followed by fluorochrome-conjugated secondary Abs as needed. The cells were analyzed using a BD FACSAria II Flow Cytometer. Each measurement contained a defined number of 2 × 10^5^ cells. Data were analyzed using BD FACSDiva and FlowJo software. Sorted cells used for qPCR analysis were stained using Itgam (CD11b) and Ly6g, and the GFP^+^CD11b^+^Ly6g^–^ populations were sorted into RNAlater (MilliporeSigma). Cells were stored at –80°C, until they were thawed for use. Cells were spun down at 500*g* in 5 mL sterile PBS, and then the pellet was resuspended in TRIzol reagent (Ambion).

### Statistics.

Data were graphed using GraphPad Prism (version 5). Student’s unpaired 2-tailed *t* test was used for comparison of 2 experimental groups. Multiple comparisons were done using 1-way and 2-way ANOVA where appropriate, followed by Tukey’s post hoc test for multiple pairwise examinations. *P* values of less than 0.05 were considered significant. Data are presented as mean ± SEM.

### Study approval.

All experiments were conducted in accordance with the NIH *Guide for the Care and Use of Laboratory Animals* (National Academies Press, 2011) and with the approval of the Virginia Tech Institutional Animal Care and Use Committee (no. 18-116; 21-044) and the Virginia-Maryland College of Veterinary Medicine, Blacksburg, Virginia, USA.

## Author contributions

EAK and ES are co–first authors. Authorship order was determined because EAK performed additional research and drafted the manuscript, while ES performed research, made figures, and wrote and edited the final version of the manuscript. EAK, ES, CK, EKGB, JJ, JL, KJP, AMK, AC, AH, CDJ, HD, GC, RDH, and XW performed research and analyzed data. ES, EAK, AMP, MLO, and MHT wrote and edited paper, designed research, and contributed reagents/analytic tools.

## Supplementary Material

Supplemental data

## Version 1 06/23/2022

In-Press Preview

## Version 2 08/08/2022

Electronic publication
