# Supplementary material for: Monocyte proinflammatory phenotypic control by ephrin type A receptor 4 mediates neural tissue damage
Source: JCI Insight. 2022 Aug 8;7(15):e156319. doi: 10.1172/jci.insight.156319 (PMC9462496; doi:10.1172/jci.insight.156319)
Supplement: Supplemental data [file jciinsight-7-156319-s135.pdf]

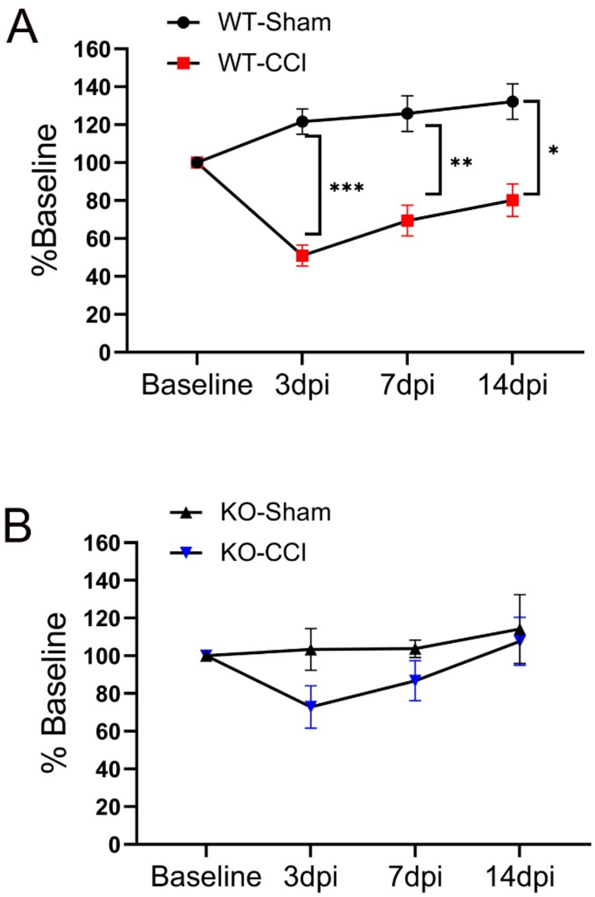

Supplemental Figure 1: Rotarod assessment of motor function shows deficits in WT but not KO mice after CCI injury. (A) +WT^{BMC} CCI-injured mice show significant reduction in motor function at 3-14dpi (n=8) compared to sham-injured mice (n=5). (B) +KO^{BMC} CCI-injured mice did not show any difference in motor function at 3-14dpi (n=10) compared to sham-injured mice (n=5). Two-way ANOVA repeated measures with multiple comparisons test. *p=<0.05; **p=<0.01; ***p=<0.0001.

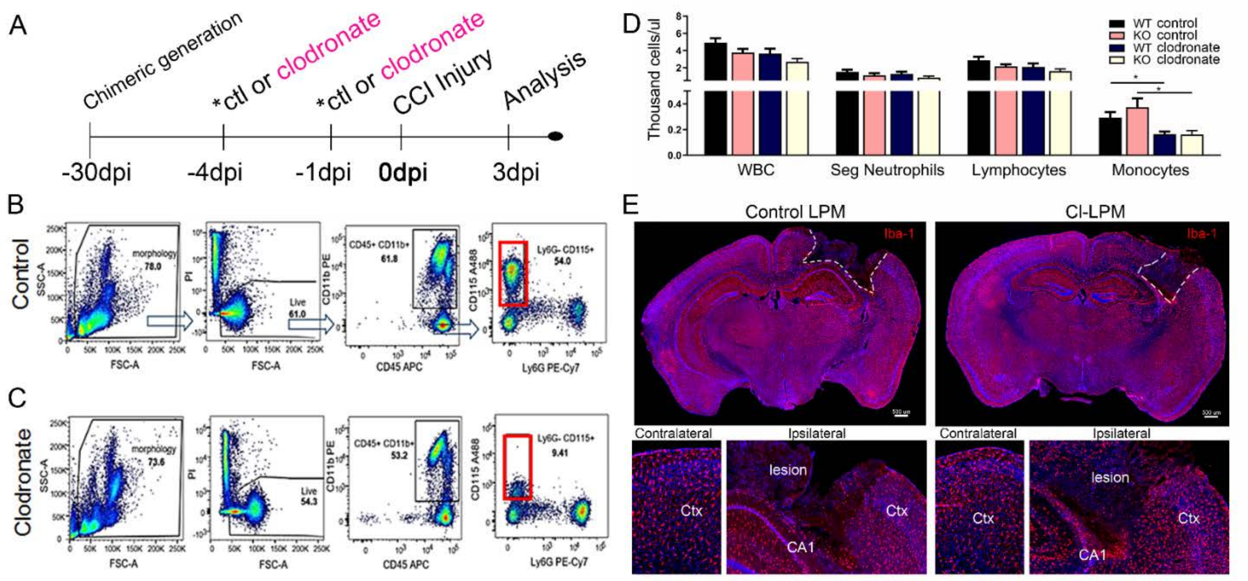

Supplemental Figure 2: Clodronate liposome treatment reduces circulating monocytes. (A) Experimental time line for peripheral monocyte depletion using clodronate liposome. CD1 male mice received 2 bolus intravenous (i.v.) injections of liposome encapsulated clodronate or control liposomes (ctl). Flow cytometry analysis was performed on peripheral blood at 24hrs. (B-C) Flow cytometry gating strategy to select single, live, CD45+, CD11b+, monocytes (CD115+/Ly6G-) and neutrophils (CD115+/Ly6G-) in peripheral blood of liposome control (B) and clodronate liposome (C) treated mice 3dpi. (D) Total count white blood cells (WBC), Neutrophils, Lymphocytes, and monocytes from +WT ^{BMC} (WT) and +KO ^{BMC} (KO) with and without clodronate treatment using complete blood counts (CBC) 24 hrs after the last injection. (E) Representative low and high magnification confocal images from control vs Cl-LPM CCI-injured brain and peri-lesion show no overt difference in microglial density in the contralateral and ipsilateral cortex at 1dpi. (F) Representative gating of Live, GFP/CD11b, Ly6G, Cx3cr1/CCR2 and Ly6C from flow sorted and stained cells isolated from the brain at 3dpi.

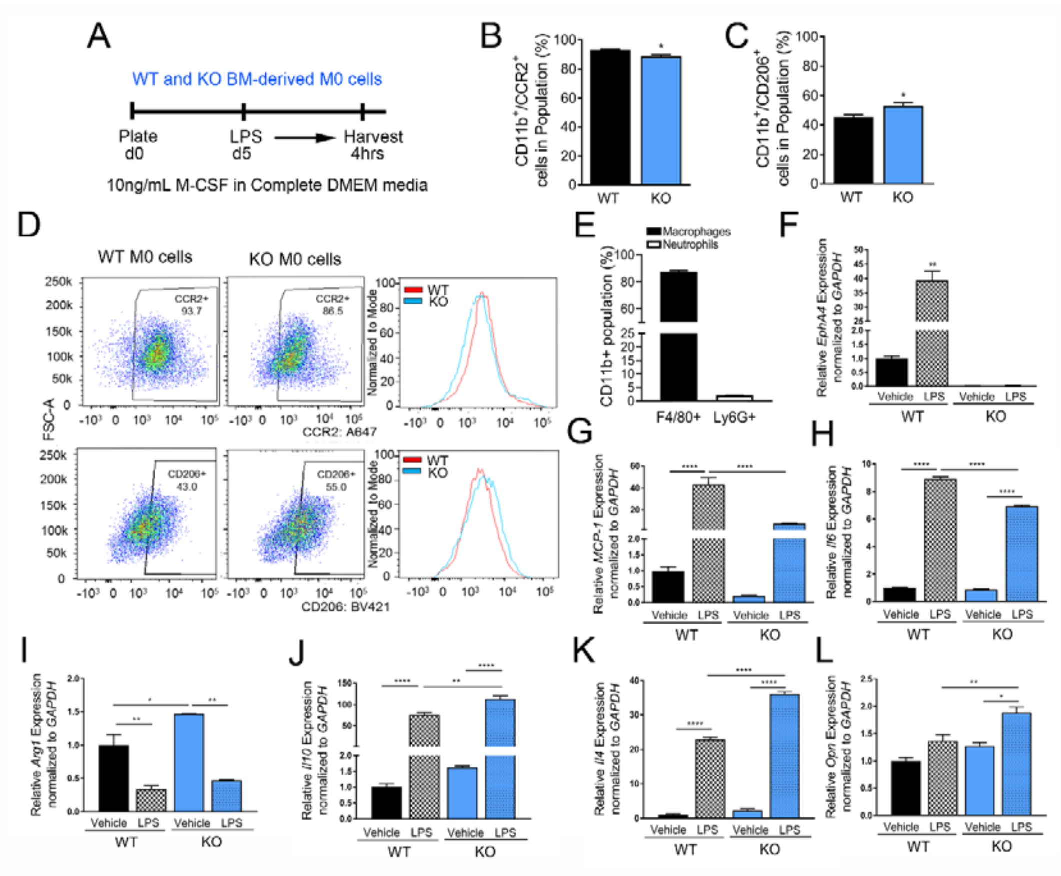

Supplemental Figure 3: EphA4 deficiency in monocytes/macrophages reduces pro-inflammatory and increases anti-inflammatory gene expression after LPS stimulation. (A) Bone marrow cells were isolated from EphA4-WT and EphA4-KO mice were cultured in complete RPMI media containing 10ng/ml M-CSF for 5 days. Bone marrow-derived monocytes (BMDMs) were then treated with LPS and harvested after 4 hours for flow cytometry and qRT-PCR analysis. (B-C) Flow cytometry analysis of the percentage of CD11b+/CCR2+ (B) and CD11b+/CD206+ (C) cell populations. (D) Representative flow cytometry plots for CCR2 and CD206 expression in WT and KO BMDMs after LPS stimulation. (E) Flow cytometry data showing that more than 80% of CD11b+cells are macrophages (F4/80+) and less than 2% are neutrophils (Ly6G+). (F-L) qRT-PCR analysis for *EphA4* (F), *MCP-1* (G), *Il6* (H), *Arg1* (I), *Il10* (J), *Il4* (K), and *OPN* (L) mRNA expression. Data are representative of at least three independent experiments. Statistical analysis was performed using Two-way ANOVA with multiple comparisons test. *p<0.05 **p<.01 ***p<0.001 ****p<0.0001

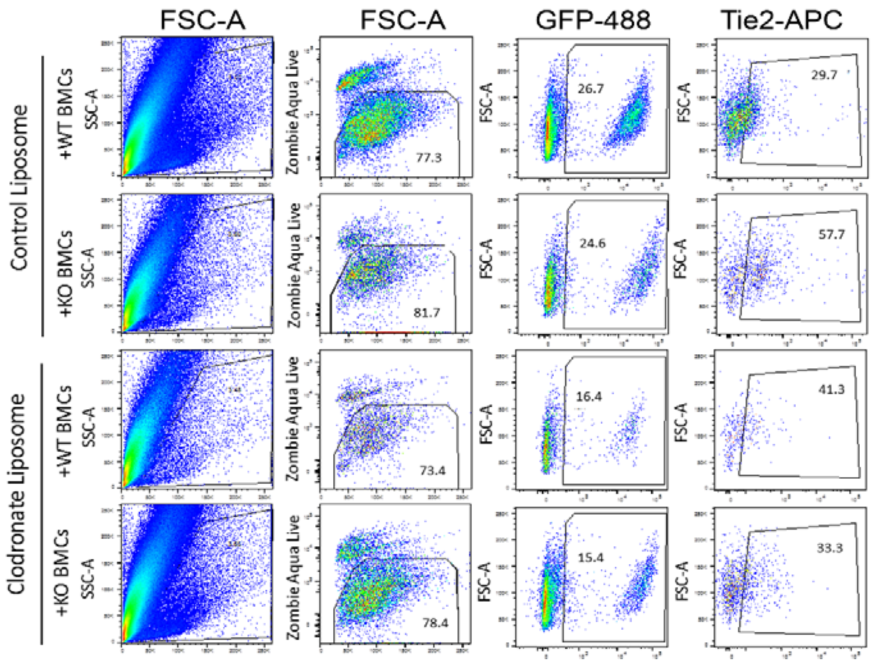

Supplemental Figure 4: Tie2 expression on infiltrating GFP-positive peripheral-derived immune cells. Representative flow cytometry plots showing the percentage of single/live/GFP+/Tie2+ cells in the ipsilateral cortex of liposome control and clodronate liposome treated +WT ^{BMC} and +KO ^{BMC} mice at 3dpi. n=5 mice per group were used for flow experiments with Tie2.
